# Effect of Drinking Water Sources on the Health of Children Under Five in South Sudan

**DOI:** 10.7759/cureus.81829

**Published:** 2025-04-07

**Authors:** Emmanuel Pitia Zacharia Lado, Japheth Osotsi Awiti, Daniel Mwai

**Affiliations:** 1 Department of Economics, University of Juba, Juba, SSD; 2 Department of Economics, University of Nairobi, Nairobi, KEN

**Keywords:** diarrhea, endogeneity, south sudan, two-stage residual inclusion, water source

## Abstract

Introduction: South Sudan has higher rates of under-five morbidity and mortality compared to other countries in the African region. Diarrhea is one of the major causes of death among children under five, both globally and in South Sudan. One of the main factors contributing to diarrheal infections, especially among young children, is the source of drinking water. This paper aims to establish the effect of drinking water sources on diarrheal morbidities among children under five in South Sudan.

Methods: Using the Second South Sudan Household Survey data, the study employed a logistic regression model to gauge the effect of drinking water sources on the health of under-five children in South Sudan. In the investigation, under-five child health was proxied by diarrheal infection in under-five children. To account for potential endogeneity and unobserved heterogeneity in the model, the research employed the two-stage residual inclusion (2SRI) procedure and control function approach, respectively.

Results: The outcomes show that out of the sample of 6,307 children, 1,561 (24.75%) had diarrhea two weeks before the survey, and 1,567 (24.85%) belonged to households using improved drinking water sources. The logistic regression result shows that the drinking water sources variable has an average marginal effect (AME) of about -0.04 and a Z-statistic of -2.72 (P-value = 0.006). This means an improved drinking water source reduces the probability of diarrheal infections among children under five. The control variables comprising the residence of the under-five children and gender of the under-five children have been weakly significant at 10%. The residence of the under-five has a coefficient of about 0.02 with a Z-statistic of 1.65 (P-value = 0.099), while the gender of the under-five child has a coefficient of about -0.02 with a Z-statistic of -1.87 (P-value = 0.062). Since the P-values are greater than 0.05, the two variables are considered to have no effect on diarrheal infections among children under five years of age. The other control variables, namely water treatment, education of head of household, mother’s age, number of under-five children, under-five child age, wealth score, and time spent on fetching water, were all insignificant.

Conclusion: Given the result of the study, it can be concluded that drinking water sources significantly determine diarrheal illnesses among children under the age of five in South Sudan. Therefore, the population needs to use potable water sources that are protected and monitored. Furthermore, additional efforts are needed to ensure access to clean water for all citizens. Providing safe drinking water from improved sources would help reduce the prevalence of diarrheal diseases and improve overall public health in the country. The strength of the investigation is that it includes a large, diverse sample of 6,307 children from all ten of the country states with different access to water sources, ensuring the findings are generalizable to a broader population. This large sample size helps account for regional variations in water quality and infrastructure, increasing the reliability and external validity of the study's results. However, the research has limitations, highlighting the necessity for a new study utilizing updated data (when available) and a composite index between drinking water sources and sanitation.

## Introduction

Child health is important to parents as a source of happiness and for the continuation of the human race in society. The health conditions of children have an important impact on their livelihood both as children and later in life as adults. Diarrhea is one of the illnesses that claim the lives of children under the age of five globally, in Sub-Saharan Africa, and particularly in South Sudan [1]. Diarrhea is the passing of loose, liquid, or watery stool, typically more than three times a day [2].

Various risk factors cause diarrhea, which has become a major cause of malnutrition among children under five worldwide [3]. The risk factors comprise access to unsafe water sources, use of unimproved sanitation facilities, unsafe disposal of children's feces, and poor hygiene practices such as the use of unsterilized feeding utensils for infants, bottle feeding, and poor food hygiene. Other risk factors include lack of or insufficient breastfeeding and vitamin A deficiency. The incidence of diarrhea in children under five has been found to vary according to different studies. For example, one study reported an incidence of around 4.5 episodes per child per year [4], while other studies reported an incidence of about five episodes per child per year in Africa [5]. Diarrhea, cholera, and typhoid are attributed to unsafe drinking water, with the highest burden of diarrhea mostly felt by children under five years of age [6]. Diarrhea is classified as the fourth killer disease worldwide and claimed the lives of 1.5 million people globally in 2019 [6].

The Sustainable Development Goals (SDGs) were established in 2015 and were meant to modify the Millennium Development Goals (MDGs). Sustainable Development Goal 3, Target 3.2, advocates for a decrease in the under-five mortality rate (U5MR) to at least as low as 25 per 1000 live births by 2030. The World Health Organization (WHO) states that although a substantial reduction in the U5MR to 38 deaths per 1000 live births was achieved globally in 2021, the African Region still had 72 deaths per 1000 live births among under-five children [1]. The chances of survival and death depend on where the under-five child is born or lives on the globe. For example, the European Region recorded only eight deaths per 1000 live births among under-five children in the same period (i.e., the year 2021). Yet in 2021, South Sudan had 99 deaths per 1000 live births among the age group, which is the highest in the region. Most of the deaths in children under five years of age are caused mainly by malaria, respiratory infections, and diarrhea [1].

Globally, only 74% of the population has access to safely managed (improved) drinking water. However, two billion people have been unable to access improved drinking water sources and relied on unimproved drinking water sources in 2020 [1]. According to the World Health Organization [7], improved drinking water is needed for all household uses, including drinking, cooking, washing hands, dishes/utensils, and personal hygiene. Improved drinking water sources include piped water into the household, yard, or plot; public tap or standpipe; tubewell or borehole; protected dug well; protected spring; and rainwater collection [7,8]. Unimproved sources of water consist of unprotected dug wells, unprotected springs, carts with small tanks or drums provided by water vendors, tanker truck provisions of water, and surface water consisting of rivers, dams, lakes, ponds, streams, canals, irrigation, and channels [7].

Sub-Saharan Africa has been the region with the highest number of people using contaminated water. About 53 percent of the people in this region use fecally polluted water without purification [8]. This is in contrast to SDG 6, Target 6.1 of the United Nations [9], which urges the consumption of clean water and improved sanitation. The Second South Sudan Household Survey (SHHS 2) established that only 69 percent of the households used improved drinking water sources. However, the 69 percent is far below the SDG 6 target 6.1 of the United Nations. Still, with 69 percent having access to improved drinking water sources, other states had lower percentages of access to improved water sources than the average reported for the country.

Most people in South Sudan use drinking water transported by trucks, carts, wells that are not protected, streams, or rivers [10]. The use of such sources of drinking water is contrary to the World Health Organization’s (WHO) guidelines [7]. This could likely become the source of illnesses, especially among the under-five children [6]. The country experiences heavy rainfall during the rainy season, which washes human and animal feces into the River Nile and other unprotected sources. Fecal coliforms in the waste products contaminate the water [11].

Gore, Wu, and Tang [12] contended that 60% of the population does not use safe drinking water, forcing this segment to rely on unsafe sources. Consequently, approximately 40% of the population enjoys access to safe drinking water. For instance, in Juba City, which doubles as the capital of Central Equatoria State (CES) and of the Republic of South Sudan, only about 20% of the residents receive piped water. The remaining households depend on water vendors utilizing truck tankers, carts, and sources such as unprotected dug wells, streams, and the River Nile [12]. The prevalence of waterborne diseases, including cholera and diarrhea, may be attributed to this issue, significantly affecting the population, particularly children under the age of five, and representing a critical public health challenge in the country.

Sustainable Development Goal 6, Target 6.1, advocates for universal and equal access to safe and affordable drinking water for all by 2030 [9]. South Sudan fell far behind the set target of Millennium Development Goal (MDG) 7 in 2015 and is unlikely to reach Sustainable Development Goal (SDG) 6, Target 6.1 by 2030, given that only a few years are left. Yet, about 64% of the population in South Sudan practices open defecation, particularly in rural areas, where 70% of them practice open defecation [10]. Such open defecation is likely to contaminate drinking water sources and can translate into diarrheal infections among the population, including children under the age of five, in the country.

According to the Second South Sudan Household Survey (SHHS 2), 30 percent of children under five had diarrhea in the two weeks preceding the survey [10]. This roughly amounts to 2,502 children under five who were found to have suffered from diarrheal infections, or approximately one out of three children under five having diarrhea.

Different studies have been carried out on the effect of water sources on diarrheal morbidities among children under the age of five. However, no similar investigation has linked the study to SDGs 3 and 6 in South Sudan. Therefore, this study aims to assess the effect of drinking water sources on the health of children under five and tries to link SDG 3 Target 3.2 to SDG 6 Target 6.1.

The study has contributed to establishing the benefit of improved drinking water sources on the health of children under five by reducing diarrheal infections. This benefit can be enhanced by promoting SDG 6 Target 6.1 and corroborated by Target 6.2 of the same SDG.

## Materials and methods

Conceptual framework

**Figure 1:**
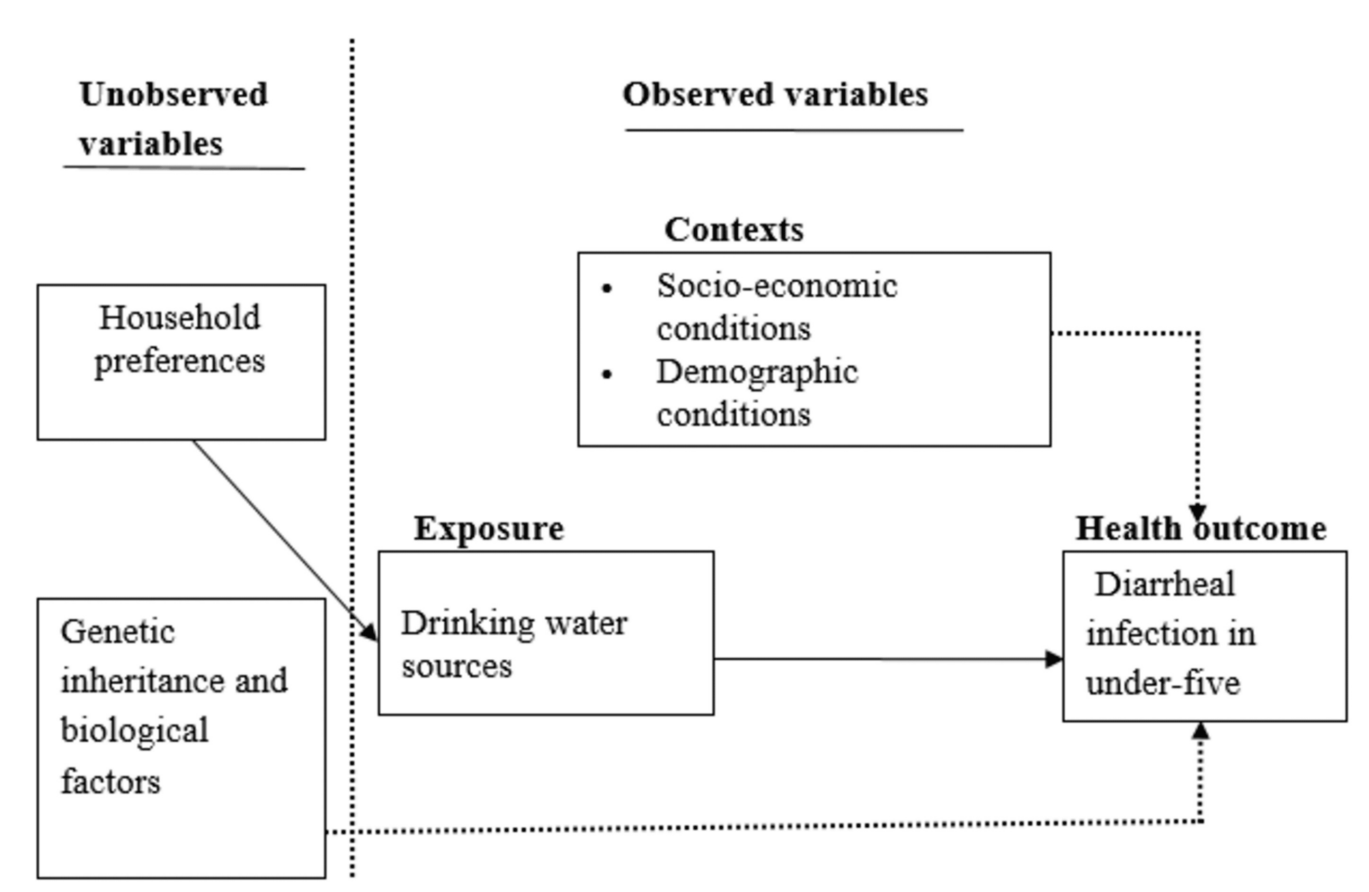
Conceptual framework of drinking water source and diarrheal infection in under-five children The image is created by the authors of this study.

The framework (Figure 1) explains the linkages between different factors and diarrhea infection in children under five. The choice of a drinking water source by a given household is determined by the preference of the household to which the child under five belongs. This is shown by the thick arrow from household preferences to drinking water source, and then the transmission of the effect of drinking water source to health outcome is equally shown by the thick arrow.

The broken arrow in the framework shows the effect of contextual factors on health outcomes in the model. These factors are control variables in the study. The contextual factors may have a confounding effect on the drinking water source's impact on diarrheal infections in children under five, or they may directly determine diarrheal infections in such children. For example, a child under five who belongs to a household with someone who has diarrhea in the same room, lives in a household with a contaminated environment where flies are present, or has eaten contaminated food, where the source of contamination is different from that of the drinking water source, can suffer from a diarrheal infection. In addition, open defecation in the community could spread diarrheal germs through feet and flies. This could cause a diarrheal infection among children under five years old, regardless of whether they live in households using water from unimproved collection points or improved sources. Parental genes and biological factors may also influence child health, as indicated by the broken arrow from genetics/biological factors to health outcomes (Figure 1). The genes of a child and biological factors can also complicate the impact of drinking water sources on diarrheal infections in children under five years of age or directly contribute to instances of diarrheal infections among children in the group.

Theoretical framework of child health

Child health can be understood using a theoretical framework in detail based on the works of Schultz (1982) [13] and Ajakaiye and Mwabu (2007) [14].

Empirical model

The health condition (H) of a child under five is represented by a diarrheal infection (DI) that occurred two weeks before the survey. The estimated effect of drinking water source and a host of control variables on diarrhea infection among children under five is presented in equation (1) below:

$DI_i = f(\text{DWS}, \text{Re}, \text{WT}, \text{Educhh}, \text{SexU5}, \text{Mage}, \text{Cage}, \text{WS}, T, e_1) \tag{1}$

Where DI, as above, is the diarrheal infection and is the outcome variable, DWS is the drinking water source variable, which is the main predictor. The rest of the variables are the control variables in the model comprising Re = residence of the given under-five child, WT = water treatment at home, Educhh = educational level of the head of the household, and sex-U5 = gender of the under-five child. The other variables in the model include mage = mother’s age in years, cage = child’s age in months, WS = wealth score, T = time spent on a round trip fetching water from the source, and $e_{1}$ = Error term.

Diarrhea is a categorical variable with two categories coded as one if an under-five child suffered from diarrhea two weeks before the survey, and zero otherwise. Given that diarrhea is binary, a logistic model is employed in the study.

In the study, drinking water source (DWS) has been the covariate of interest (as stated earlier) and was coded as one if a given household where the under-five child lives or is born was using water from an improved source, and zero otherwise.

DWS was assumed to be endogenous in the model, given the assumption of reverse causality between diarrheal infection and drinking water source. Endogeneity could have equally resulted from errors in the measurement of DWS, which is the covariate of interest. In this study, the two-stage residual inclusion (2SRI) procedure was adopted to take control of potential endogeneity in the model [15-18]. The study used the proportion of households using improved drinking water sources (PHUIDWS) as an instrumental variable. Drinking water source (DWS) has also been considered as a categorical covariate of a binary nature: whether DWS was improved was coded as 1, or 0 otherwise.

DWS has to depend on PHUIDWS at the cluster level, which is the instrumental variable in the study and the host of the control variables as in equation (1).

Estimating equation (2) below using a logistic regression model, given the binary categorical nature of DWS, yields the results of the first-stage regression model:

$DWS_i = f(\text{PHUIDWS}, \text{Re}, \text{WT}, \text{Educhh}, \text{SexU5}, \text{Mage}, \text{Cage}, \text{WS}, T, e_2) \tag{2}$

where $e_{2}$ is the error term in the first-stage regression model in equation (2) above. After estimating equation (2) above, the generalized residuals - $\hat{e}_{2}$ - were extracted and included as a separate covariate in equation (1) to control for endogeneity [16,18].

To control for unobserved heterogeneity, an interactive term, which is the product of the drinking water source (the assumed endogenous covariate of interest) and the generalized residuals from the first-stage regression, $DWS*\hat{e}_{2}$, was added to the second-stage regression model to form equation (3) [16-18].

$DI_i = f(\text{DWS}, \text{Re}, \text{WT}, \text{Educhh}, \text{SexU5}, \text{Mage}, \text{Cage}, \text{WS}, T, \hat{e}_2, \text{DWS} \cdot \hat{e}_2, e_1) \tag{3}$

Since diarrheal infection is a binary variable, the study adopted a logistic regression model with the assumption that the error term, $e_{1}$, is logistically distributed [19]. Using a logistic model or a probit model does not create any differences in terms of significance, as the coefficients of each of the two can be transformed into the coefficients of the other by using a weight [16,19].

Data

The data used in the study were extracted from the Multiple Indicator Cluster Survey (MICS) of the Second South Sudan Household Health Survey (SHHS 2) in 2010. The SHHS 2 was conducted as a component of the fourth global round of MICS surveys (MICS4). The datasets were accessed through the UNICEF global database at (https://mics.unicef.org/surveys). The sample size covered in the MICS survey was 8,338 under-five children. The information was extracted from mothers or caretakers of children under five. The survey was meant to assess the well-being of mothers aged 15-49 years and children aged 0-59 months to inform strategies towards attaining the MDGs at that time. The MICS dataset provides a basis for comparison of the situation of children and women in South Sudan in terms of health and general well-being versus other countries.

Model identification

The study employed one covariate of interest and a host of control variables in the model. Since the independent variable of interest is only one, there has to be at least one instrumental variable [20]. Given the fact that one instrumental variable was employed for one covariate in the study, the model became just identified [20].

To take care of endogenous effects in the drinking water sources, an instrumental variable technique was used. Since the study made use of a limited dependent variable in the context of the logistic model, the 2SRI procedure was used here [15,16,20,21]. The use of 2SRI under the logistic regression model has been employed to take control of endogeneity [21].

This study employed the proportion of households using improved drinking water sources at the cluster level to instrument the drinking water sources. The choice has been based on logical and statistical grounds.

On logical grounds, a high proportion of improved drinking water sources at the cluster level is likely to increase the probability of a household within the cluster using improved drinking water sources. If a household moves to a residence closer to improved drinking water sources within the cluster, it will not affect the behavior of the other households in the country. Furthermore, if the household's movement closer to the drinking water sources is within the same cluster, it will not affect the proportion of households using improved drinking water sources. However, the proportion of improved drinking water sources at the cluster level can affect the probability of a household using an improved drinking water source within that cluster. Therefore, the problem of endogeneity in this instrumental variable is unlikely to exist.

Empirically, the choice for the proportion of households using improved drinking water sources at the cluster level was based on Awiti & Wooldridge [16,20] (i) the exogeneity of this variable in the main equation i.e. $Cov\left( PHUIDWS,e_{1} \right)=0$ (ii) The proportion of households using improved drinking water sources at the cluster level is a relevant instrumental variable since it has a significant effect on the endogenous independent variable of interest. That is $Cov(DWS,PHUIDWS)\neq 0$.

An increase in the proportion of households using drinking water from improved sources at the cluster level is likely to increase the likelihood of a household within that cluster using improved drinking water sources.

Estimation issues

In this study, diarrheal infection in children under five was the outcome variable, and the drinking water source variable was the regressor of interest. Additionally, control variables were included to account for confounding effects in the model. Precisely, the covariate considered in this model is drinking water sources (DWS), along with control variables for potential confounders whose effects may confuse the relationship between drinking water sources and diarrheal infections among under-five children.

Potential endogeneity

In estimating the effect of drinking water sources on diarrhea infection, care has to be taken to control for endogeneity in the model. The simultaneity between drinking water sources and diarrhea, the unobserved cases of confounding, and the errors of measurement in the covariate of interest are likely to cause endogeneity in the model. Estimating a model in the presence of endogeneity would lead to biased and inconsistent results [15-17,21]. To control for endogeneity, the study employed the 2SRI procedure to remove potential endogeneity effects in the model. The 2SRI procedure is suitable for controlling endogeneity in nonlinear models [15-18,21]. This is achieved by estimating the first-stage regression model, extracting generalized residuals, and including these residuals as a separate regressor in the second-stage regression model [16,18]. If the coefficient of the generalized residuals’ term in the diarrhea model is significant, then the drinking water sources are endogenous in the model; otherwise, they are not [16].

Unobserved heterogeneity

Unobserved heterogeneity needs to be addressed to obtain unbiased results regarding the effect of drinking water sources on diarrhea infection in children under five. This unobserved heterogeneity can stem from factors that are not observed and may interact with the drinking water sources in the model for diarrhea. This interaction may potentially lead to an erroneous effect of the water sources on an under-five child's health in the model. In addition, the unobserved heterogeneity may arise from differences in environmental risk conditions in which the children live, differences in genetic or biological factors across children, unexplained differences in socioeconomic components, and differences in the level of care the children receive from their parents and relatives [22]. These variations across children may be known to the mothers or households to which the children belong but remain unknown to the researcher. To control for unobserved heterogeneity in the model, the study made use of the control function approach [16-18]. This is done by multiplying the generalized residuals from the first-stage regression model and the endogenous regressor of interest and including this interaction term as one of the independent variables in the model. 

Definitions and measurements of variables

In the study, the variables that entered the model are presented in Table 1 below.

**Table 1:** Definitions and measurements of variables

Variable	Definition and measurement
Under-five suffered diarrheal infection	1 if an under-five child suffered from diarrheal infection two weeks before the survey was conducted, 0 otherwise.
Improved drinking water source (DWS)	1 if the drinking water source was improved, 0 otherwise.
Urban residence	1 if the under-five child resides in an urban area, 0 otherwise.
Water treated	1 if water was treated at the household level, 0 otherwise.
Head of household attained secondary education, and above.	1 if the head of the household reached secondary level or above, 0 otherwise.
Male under-five child	1 if the under-five child was male, 0 otherwise.
Mother’s age	Mother’s age is measured in years and is a continuous variable.
Number of under-five children	Total number of under-five children who live together in the given household as a continuous variable.
Under-five child age	Under-five child age in months and is a continuous variable.
Wealth score	Wealth score is a measure of household economic conditions and is a continuous variable.
Time of fetching water is more than 30 minutes round trip	1 if time spent in round trip fetching water is greater than 30 minutes, 0 otherwise.
Proportion of households using improved drinking water sources at cluster level	Proportion of households using improved drinking water sources at the cluster level (PHIDWS) is the instrumental variable for drinking water sources and is a continuous variable.
Generalized residuals	The generalized residuals from the first-stage regression (drinking water source) model.
Interaction term	The interaction of the generalized residuals of the drinking water sources and the drinking water sources.

## Results

A total of 6,307 mothers and caregivers provided complete responses in the South Sudan Household Survey 2, which were subsequently utilized in the study (Table 2). The findings revealed that 24.75% of children under five experienced diarrhea within the two weeks preceding the survey, whereas 75.25% were unaffected by diarrhea. In terms of drinking water sources, about 24.85% of the children belonged to households that accessed improved drinking water, while 75.15% sourced their water from unimproved facilities. The research indicated that only 27.18% of the under-five children lived in urban settings, in contrast to 72.82% who resided in rural areas. In relation to water treatment practices, 18.77% of the children originated from households that implemented home water treatment methods, whereas 81.23% did not participate in any water treatment activities at their homes.

**Table 2:** Frequency and percentage of categorical determining factors of under-five diarrheal infection (n = 6,307) Note: The determining factors that are not categorical were excluded from Table 2.

Variable	Frequency (n)	Percentage (%)
Diarrheal infection		
Child suffered diarrheal infection	1,561	24.75
No diarrheal infection	4,746	75.25
Drinking water sources		
Improved	1,567	24.85
Unimproved	4,740	75.15
Residence		
Urban	1,714	27.18
Rural	4,593	72.82
Water treatment		
Treated	1,184	18.77
Untreated	5,123	81.23
Educational level of head of household		
Secondary level and above	1,535	24.34
Below Secondary level	4,772	75.66
Gender of the under-five child		
Male	3,271	51.86
Female	3,036	48.14
Time spent in a round trip on fetching water		
More than 30 minutes round trip	4,117	65.28
30 minutes or less	2,190	34.72

The analysis revealed that out of the total number of heads of households, only 24.34% of the individuals had attained an education level of secondary school or higher. In contrast, a significant majority, comprising 75.66%, had educational qualifications below the secondary level. When examining the population of children under five years of age by gender, it was noted that the number of male children slightly exceeded that of female children in the study. Specifically, 51.86% were males while 48.14% were females. In terms of the duration required for a round trip to collect water from designated sources, it was observed that 65.28% of the households took more than 30 minutes for this task, whereas 34.72% of them completed the round trip in 30 minutes or less.

In reporting the regression results, economists prefer reporting average marginal effects (AMEs) instead of marginal effects at the mean [16,23]. When the independent variable is a dummy in nature, discrete changes are computed instead of marginal effects [23].

The marginal effect at the mean (MEM) is limited given that [24] the logistic regression model is nonlinear, making it difficult to convert MEM into a change in predicted probability for a discrete change in the covariates. The average of a regressor may not correspond to any known values in the data, and as such, it is reasonable to average the observations. MEM is not suitable for any regressors that are binary [24]. It is argued that MEM is only appropriate when a covariate is formed from continuous covariates in the model.

The results of the first-stage regression model are presented in Table 3. As seen in Table 3, the proportion of households using improved drinking water sources at the cluster level increases the probability of a household within the cluster using drinking water from an improved source by approximately 0.27, with everything else remaining constant. All the control variables in the first-stage regression, except water treatment at home, male under-five children, the number of under-five children in a given household, the age of the indexed child, and the time spent in a round trip fetching drinking water, have effects on the drinking water sources.

**Table 3:** Average marginal effects of the proportion of households using improved drinking water sources and the drinking water sources with Z-statistics in parentheses (n = 6,307) Note: The results of the first-stage regression model with Z-statistic in parentheses. The level of significance is: *** = p< 0.001, ** = p< 0.05.

Covariate	Average marginal effects	P-value
Proportion of households using improved drinking water sources at cluster level	0.2693759*** (14.47)	0.000
Urban residence	0.0204635 (1.69)	0.091
Water treated at the household level	0.0141891 (1.06)	0.291
Head of household attained secondary education, and above	-0.1032961*** (-7.45)	0.000
Male under-five child	-0.0021359 (-0.20)	0.840
Mother’s age in years	0.0012955** (2.05)	0.040
Number of under-five children in a household	-0.0108515 (-1.63)	0.103
Child's age in months	-0.0002704 (-0.80)	0.424
Wealth score	-0.0349823*** (-5.28)	0.000
Time spent on a round trip fetching water from the source	0.0153159 (1.37)	0.171

Table 4, column (1) contains the covariates, column (2) contains the results of the baseline model with Z-statistics in parentheses, and column (3) contains the p-values. Column (4) hosts the results of the 2SRI with their Z-statistics in parentheses, while column (5) is for the p-values. Column (6) has the CFA results with the Z-statistics in parentheses and the p-values in column (7). Since the study results are reported in terms of average marginal effects (AMEs), the Z-statistics are reported alongside the AMEs without the t-statistics. In particular, column 2 contains AMEs of the logit (baseline) model, column (4) contains the AMEs of the 2SRI model that controls for potential endogeneity, and column (6) contains the AMEs of the control function approach (CFA) that is used to mitigate the effect of unobserved heterogeneity in the model. As can be seen, the coefficient of the generalized residuals has been insignificant. This means the model does not suffer from endogeneity. Estimating equation (1) produces the baseline (logit) model results in Table 4, column 2.

**Table 4:** Average marginal effects of drinking water source and diarrhea infection with Z-statistics in parentheses (n = 6,307) Note: The results of the baseline model are presented in column (2), the results of the model controlling for endogeneity in column (4), and the results of the model controlling for both endogeneity and heterogeneity in column (6). The Z-statistics are in parentheses. Level of significance: *** = p< 0.001, ** = p< 0.05. CFA: control function approach; 2SRI: two-stage residual inclusion

(1) Covariate	(2) Logit (baseline) model	(3) P-value	(4) 2SRI	(5) P-value	(6) CFA	(7) P-value
Improved drinking water sources	-0.0352821*** (-2.72)	0.006	-0.1423397** (-2.07)	0.039	-0.1712003 (-1.89)	0.059
Urban residence	0.0203763 (1.65)	0.099	0.0223756 (1.80)	0.072	0.0222615 (1.79)	0.073
Water treated	-0.0116189 (-0.82)	0.411	-0.0095031 (-0.67)	0.503	-0.0096873 (-0.68)	0.495
Head of household attained secondary education, and above	-0.0201795 (-1.54)	0.123	-0.0320721** (-2.13)	0.033	-0.0312151** (-2.06)	0.039
Male under-five child	-0.0202701 (-1.87)	0.062	-0.0203166 (-1.87)	0.061	-0.020423 (-1.88)	0.060
Mother’s age in years	-0.0005447 (-0.84)	0.403	-0.0003855 (-0.59)	0.558	-0.0003806 (-0.58)	0.563
Number of under-five children in a household	-0.0047076 (-0.70)	0.487	-0.0056252 (-0.83)	0.408	-0.0056361 (-0.83)	0.407
Child's age in months	0.0003576 (1.03)	0.302	0.0003302 (0.95)	0.341	0.0003293 (0.95)	0.343
Wealth score	-0.0058274 (-1.11)	0.266	-0.0093291 (-1.64)	0.101	-0.0090242 (-1.58)	0.115
Time spent on a round trip in fetching water from the source	0.0000509 (-0.00)	0.996	0.0018402 (0.16)	0.873	0.0017493 (0.15)	0.879
Generalized residuals from drinking water sources			0.1106001 (1.58)	0.113	0.0921981 (1.17)	0.243
Drinking water sources interaction with generalized residuals					0.0651394 (0.49)	0.621
Wald Test: x^2^ (10)	18.63**	0.0452				
Number of observations (N)	6,307		6,307		6,307	

Furthermore, the coefficient of the interactive term between the generalized residuals of the drinking water sources and the drinking water sources has been statistically insignificant. This means that the model does not have any heterogeneity. Therefore, the study considers the results of the Logit (baseline) model in column 2 of Table 4 for interpretation and conclusions. In addition, the Wald Chi-square (10) is 21.34, which is a test for the model’s fitness, and has been statistically significant at the 5% level (column 2 of Table 4). The significance of the Wald Chi-square statistic shows that the model is well-fitted. The study suspected the presence of collinearity between drinking water sources and water treatment at the point of use (home). A test of correlation using a correlation matrix was conducted, and the result showed that the correlation coefficient between the two variables was 0.02, which is nearly 0 [20]. This means that there has been no serious problem of collinearity between the two independent variables in the model. 

As seen in Table 4, the level of significance for the discrete change in the drinking water sources is 1%. The result shows that the drinking water sources (DWS) variable is a significant factor in determining diarrheal infections among children under five in the country. Keeping all other factors constant, being a child of a household using drinking water from an improved source reduces the probability of diarrheal infection in an under-five child by approximately 0.04 compared to a counterpart who lives in a household using drinking water from unimproved sources.

Since the research views values with p-values below 0.001 and 0.05 as significant, all the control variables have proven insignificant given their respective p-values. That is, the residence of the under-five child, the gender of the under-five child, water treatment, and educational level of the head of the household, the mother's age in years, and the number of under-five children in a given household were found to be insignificant in the model. Other control variables that were also found to be insignificant include child age in months, wealth score, and time spent on a round trip fetching water from the source.

As stated earlier, the coefficient of the generalized residuals, $\hat{e}_{2}$, has been statistically insignificant in the model, which indicates that the drinking water sources variable has not been endogenous in the model. In addition, the interaction term, $DWS*\hat{e}_{2}$, which is included in the model to control for unobserved heterogeneity, turned out to be equally statistically insignificant. This means that the model does not suffer from heterogeneous effects.

## Discussion

The decrease in the probability of diarrheal infection among children under five who belong to households using improved drinking water sources could be due to the prevention of contaminated drinking water sources. The finding of the effect of drinking water sources on diarrheal infections in under-five children is consistent with the results established in studies conducted in different countries [5]. Other studies that have confirmed the significance of improved drinking water sources in reducing diarrheal infections among children under five years old include studies by Acharya et al. [2] & Geere & Hunter [25]. A positive behavioral change in all households towards using improved drinking water sources would save the country’s population, especially the under-five children, from diarrheal incidences. This would reduce diarrheal morbidities and, hence, reduce mortalities among children under five. Behavioral change must be achieved through public and/or community awareness regarding protecting and monitoring drinking water sources. The enlightenment programs can be through radio, TV, newspapers, text messages, and water management committees (WMC). The households that lack access to improved drinking water are advised to boil water from unimproved sources before use.

Contrary to the above findings, some studies have failed to establish a significant effect of drinking water sources on diarrheal infections among the age group [26]. Given these findings, children under the age of five, among others, are likely to experience diarrheal infections if drinking water sources are not improved and made readily accessible to the population in South Sudan. To prevent diarrheal incidences in the country, drinking water sources have to be preserved from contaminants. Additionally, there is a need to raise awareness among the country's population about the dangers of fetching drinking water from contaminated sources. Unimproved drinking water sources and/or water points should be avoided as much as possible to protect humans, especially young children, from diarrheal infections. There is a need for a concerted effort towards creating an enabling environment for every household to have clean drinking water to attain SDG 6, Target 6.1. This would contribute towards achieving SDG 3, Target 3.2 in South Sudan. That means policies have to be formulated and enforced by law enforcement agencies, with penalties imposed on those who contravene the rules of no open defecation. In addition, communities need to be encouraged to construct latrines/toilets by offering incentives. For example, food for work is provided to those who have constructed improved sanitation facilities in their homes. Above all, the government has to invest in a safely managed water infrastructure.

The study has successfully achieved its goal based on the findings. The study has its strengths, given a large, diverse sample of 6,307 children from various states with different access to water sources, ensuring the findings are generalizable to a broader population. This large sample size helps account for regional variations in water quality and infrastructure, increasing the reliability and external validity of the study's results. However, it is not without its limitations. First, the research relies on data from a survey conducted in 2010, which may not reflect subsequent changes in the country. Therefore, it is advisable to conduct a follow-up study utilizing updated MICS data, should a new survey be available. Second, the impact of water on human health cannot be adequately assessed in isolation from sanitation. Due to concerns regarding collinearity between drinking water sources and sanitation facilities within the model, the sanitation variable was excluded. Future research should consider employing a composite index that integrates drinking water sources and sanitation facilities rather than relying on a single variable.

## Conclusions

The study identified drinking water sources as a determining factor for diarrheal infections among children under the age of five years in South Sudan. This means that the management and protection of drinking water sources must be taken seriously to reduce diarrheal infections in children, especially those under the age of five. It has been established that the proportion of access to clean drinking water sources and improved sanitation facilities in the country is only six percent. Implementing a policy to protect sources of drinking water requires educating the public about the dangers of open defecation and other pollutants. This means that law enforcement agencies must create and implement policies, and those who violate the prohibition on open defecation face serious consequences. Incentives must also be provided to communities to encourage the construction of toilets. For example, the government should provide food for work to individuals who have constructed improved sanitation facilities in their homes. The government must, above all, make investments in water infrastructure that is safely managed. There is a need for clean drinking water to be made readily accessible to all households as per SDG 6, Target 6.1, to contribute towards the attainment of SDG 3, Target 3.2, in the country. In the interim, households that lack access to improved drinking water are advised to boil water from unimproved sources before use. Behavioral change must be achieved through public and/or community awareness regarding protecting and monitoring drinking water sources. The enlightenment programs can be through radio, TV, newspapers, text messages, and workshops, making it one of the civic education programs in schools.
